# Association of cerebral white matter lesions with cognitive function and mood in Japanese elderly people: a population-based study

**DOI:** 10.1002/brb3.315

**Published:** 2015-02-03

**Authors:** Mika Yamawaki, Kenji Wada-Isoe, Mikie Yamamoto, Satoko Nakashita, Yusuke Uemura, Yoshimitsu Takahashi, Takeo Nakayama, Kenji Nakashima

**Affiliations:** 1 Division of Neurology, Department of Brain and Neurosciences, Faculty of Medicine, Tottori UniversityYonago, Japan; 2 Department of Health Informatics, Kyoto University School of Public HealthKyoto, Japan

**Keywords:** Cognitive function, deep white matter hyperintensities, mood, periventricular hyperintensities, population based

## Abstract

**Background:**

To determine the relationships between regional white matter lesions (WMLs), lifestyle factors, and cognitive, motor function and mood.

**Methods:**

A comprehensive evaluation, including brain MRI, blood tests, the Unified Parkinson's Disease Rating Scale, the Mini Mental State Examination, and the Geriatric Depression Scale, was performed for people aged 65 years or older living in Ama-cho on October 1, 2009. Participants were classified by severity of periventricular hyperintensities (PVH) and deep white matter hyperintensities (DWMH) using the Fazekas score.

**Results:**

Of 900 eligible participants, 688 (76.4%) were enrolled, including 303 men. Significant predictors of severe PVH were older age, lower low-density lipoprotein cholesterol (LDL-C) levels, elevated blood pressure (BP), cerebral infarction, and no current alcohol use. Significant predictors of severe DWMH were older age, lower 1,5-anhydroglucitol (1,5-AG) levels, elevated BP, cerebral infarction, and no current alcohol use. Higher cognitive function was associated with younger age, female sex, mild DWMH, more years of education, and higher high-density lipoprotein cholesterol levels. Depressive symptoms were associated with lower 1,5-AG levels, lower LDL-C levels, moderate to severe PVH, and no current alcohol use.

**Conclusions:**

White matter lesions in elderly people were related to hypertension and impaired glucose tolerance. The severity of WMLs was associated with cognitive function and mood.

## Introduction

Large increases in the number of elderly people with cerebrovascular disorders and dementia in Japan have created significant economic and personal burdens. Dementias, such as Alzheimer's disease and vascular dementia, are the main causes of functional decline in the elderly, and cerebrovascular disorder is a major source of bedridden state (Yoshida et al. 2012). Prevention of these disorders is important for elderly people to maintain their ability to perform activities of daily living and a good quality of life.

Cerebral white matter lesions (WMLs) are associated with declines in cognitive, motor function, and mood (Gouw et al. 2006; Herrmann et al. 2008; Debette and Markus 2010). Risk factors for WMLs include lifestyle factors, hypertension, and renal damage (de Leeuw et al. 2002; Weiner et al. 2009). On the one hand, unlike Western countries, Japan has a relatively higher incidence and mortality caused by stroke than coronary heart disease. In addition, the dominant type of ischemic stroke in Japan differs from it in Western countries (Ueshima et al. 2008). In such the situation, we take an interest in research findings of WMLs in our country. However, there have been few epidemiological studies of these associations in Japan. Therefore, we examined the relationship between these risk factors and WMLs in Japanese elderly inhabitants in a rural island town, with very little population movement in the elderly.

## Methods

### Study Population

This study was conducted in the municipality of Ama-cho in the northwestern part of Japan (Wada-Isoe et al. 2012). The target population included all persons ages 65 years and older who had been recorded in the Basic Resident Registration of the Ama-cho on October 1, 2009. The subjects knew the recruitment of this study by the appeal of Tottori University and the town office. An exclusion criterion was the usual contraindications to MRI. The study was approved by the Tottori University committee for medical research ethics following the principles outlined in the Declaration of Helsinki, and all participants provided written informed consent.

### Data Collection

Hypertension, impaired glucose tolerance, hyperlipidemia, current smoking and alcohol use, and years of education were obtained from a patient-administered questionnaire and review of electronic databases of healthcare system. Blood pressure (BP) at evaluation was assessed during medical examinations. Concurrently with the MRI investigation, triglycerides, high-density lipoprotein cholesterol (HDL-C), low-density lipoprotein cholesterol (LDL-C), creatinine (Cr), and 1,5-anhydroglucitol (1,5-AG) were measured by us for this investigation.

### MRI and Measurement of WMLs

Brain MRI scans were performed between March 2010 and May 2010 using 1.5-T scanners (Gyroscan Intera; Philips Electronics Japan, Tokyo, Japan). The scanning protocol included a series of axial proton-density (repetition time [TR], 3000 msec; echo time [TE], 12 msec), T2-weighted (TR, 3000 msec; TE, 96 msec), and sagittal contiguous T1-weighted (TR, 8.6 msec; TE, 4 msec) images.

The Fazecas scale was used to assess periventricular hyperintensities (PVH) and deep white matter hyperintensities (DWMH) (Fazekas et al. 1987). Participants were classified into three severity groups according to their Fazekas score: mild (0–1), moderate (2), and severe (3). The presence of infarcts was assessed visually by a neurologist using a standardized assessment. WMLs were areas of bright, high-signal intensities noted on MRI T2, and proton-density-weighted images. Cerebral infarcts were defined as focal hyperintensities on T2-weighted images ≥3 mm, with corresponding prominent hypointensities on T1-weighted images.

The two neurologists (M.Y. and M.Y.) evaluated 10% of cases for degree of concordance. The inter- and intrarater kappa coefficients for PVH were 0.846 and 0.818, respectively, and for DWMH were 0.660 and 0.867, respectively.

### Assessment of Cognitive, Motor Function, and Mood

A standardized neurological examination was conducted by neurologists as previously described, which included an abbreviated (10-item) version of the motor portion of the Unified Parkinson's Disease Rating Scale (mUPDRS) (Uemura et al. 2011, 2013). The 10 items (each rated from 0 to 4) assessed speech, facial expression, tremor at rest, rigidity (rated separately in the neck, right arm, left arm, right leg, and left leg), posture, and body (axial) bradykinesia. As mUPDRS is associated with measures of physical activity obtained using actigraphy, mUPDRS provided a means to assess motor impairment (Uemura et al. 2011). The mini mental state examination (MMSE) was administered to determine global cognitive function (Wada-Isoe et al. 2012). The Japanese version of the Geriatric Depression Scale (GDS) with 15 questions was administered to assess depressive symptoms (Uemura et al. 2011, 2013).

### Statistical Analysis

The chi-square tests were used to determine significant differences of the frequencies of categories between the groups. Kruskal–Wallis and Mann-Whitney U tests were used for demographic and clinical comparisons between the groups. Multivariate logistic regressions with stepwise selection and likelihood ratio test were performed to determine the independent predictors of severe PVH and DWMH. In multivariate regression analyses, forward stepwise regression was used to determine the independent predictors of mUPDRS, MMSE and GDS. We chose variables with a *P* value of less than 0.25 in the univariate analysis as the final candidate variables for the multivariate statistics. We confirmed that predictors were not highly correlated with others. Significance was defined as *P* < 0.05. All analyses were conducted using SPSS (release 20; SPSS, Tokyo, Japan).

## Results

Of 900 eligible individuals, 689 (76.6%) were enrolled in the study. The remaining 211 participants did not undergo an MRI scan of the brain despite our eager and repeated appeals prompting their participation. One participant was excluded from analyses because of poor quality MRI due to movement. Thus, this study comprises the remaining 688 participants (76.4%). Compared with participants, nonparticipants were similar in gender, but age of nonparticipants was significantly increased compared with participants (data not shown). The sample included 303 (44%) males. The sample had a mean ± SD age of 76.5 ± 7.1 (age composition of participants: 69 years of age or younger, 19.6%; 70–79 years of age, 47.2%; 80–89 years of age, 28.9%; 90 years of age or older, 4.2%). The median score of PVH and DWMH were 1 (interquartile range, 1–2) and 1 (interquartile range, 1–2), respectively. There were no significant differences in these variables by sex. Cerebral infarctions were present in 211 participants (30.7%). Most of cerebral infarctions were regarded as small infarctions with a diameter of <15 mm.

### Predictors of PVH Severity

Table 1 summarizes participant characteristics by PVH severity. The logistic regression model included age, systolic blood pressure (SBP), Cr, HDL-C, LDL-C, 1,5-AG, current drinker, and cerebral infarction. We revealed that older age, lower LDL-C levels, elevated SBP, presence of cerebral infarction, and no current alcohol use were significant predictors of severe PVH (Table 2).

**Table 1:** Characteristics of participants by PVH severity

Characteristic	Mild (*n* = 438)	Moderate (*n* = 176)	Severe (*n* = 74)	All (*n* = 688)
Male (%)	45.2	38.6	50.0	44.0
Age, mean ± SD, years	74.4 ± 6.3	79.5 ± 6.6^1^	82.0 ± 6.9^1^	76.5 ± 7.1
SBP, mean ± SD, mmHg	133.3 ± 17.5	136.6 ± 18.6	139.9 ± 19.3^1^	134.7 ± 18.1
DBP, mean ± SD, mmHg	74.8 ± 10.3	75.2 ± 10.9	75.8 ± 9.8	75.0 ± 10.4
Cr, mean ± SD, mg/dL	0.72 ± 0.22	0.77 ± 0.35	0.87 ± 0.42^1^	0.75 ± 0.29
HDL-C, mean ± SD, mg/dL	57.0 ± 14.1	55.1 ± 13.8	53.2 ± 13.1^1^	56.1 ± 14.0
TG, mean ± SD, mg/dL	155.6 ± 107.2	147.3 ± 92.9	130.1 ± 78.0^1^	150.7 ± 101.1
LDL-C, mean ± SD, mg/dL	101.3 ± 28.5	98.9 ± 28.5	88.8 ± 29.9^1^	99.3 ± 28.9
1,5-AG, mean ± SD, *μ*g/mL	21.1 ± 8.8	19.3 ± 9.7	19.1 ± 10.0	20.4 ± 9.2
Cerebral infarction (%)	21.7	44.3^2^	51.4^2^	30.7
Hypertension (%)	60.2	70.5^2^	71.6	64.0
Impaired glucose tolerance (%)	32.9	38.1	36.5	34.6
Hyperlipidemia (%)	34.3	33.0	29.7	33.5
Current smoker (%)	8.0	8.1	10.0	8.3
Current drinker (%)	33.4	24.4^2^	22.9	30.1
mUPDRS, mean ± SD	0.8 ± 1.9	1.3 ± 1.9^1^	1.5 ± 1.8^1^	1.0 ± 1.9
MMSE, mean ± SD	26.6 ± 3.2	25.3 ± 3.8^1^	23.4 ± 5.4^1^	25.9 ± 3.8
GDS, mean ± SD	2.9 ± 2.9	3.8 ± 3.4^1^	4.1 ± 3.7	3.3 ± 3.2
Education, mean ± SD, years	9.8 ± 2.1	9.6 ± 2.2	9.1 ± 2.5	9.7 ± 2.2

Cr, creatinine; DBP, diastolic blood pressure; GDS, Geriatric Depression Scale; HDL-C, high-density lipoprotein cholesterol; LDL-C, low-density lipoprotein cholesterol; mUPDRS, motor component of Unified Parkinson's Disease Rating Scale; MMSE, mini mental state examination; PVH, periventricular hyperintensities; SBP, systolic blood pressure; SD, standard deviation; TG, triglycerides; 1,5-AG, 1,5-anhydroglucitol.

1 *P* < 0.05, Kruskal–Wallis test with post hoc test versus mild.

2 *P* < 0.05, chi-square test versus mild.

**Table 2:** Predictors of severe PVH^1^

Variable	Regression coefficient	OR (95% CI)	*P*	Predictive accuracy
Intercept	−15.600		<0.001	89.7
Age	0.148	1.160 (1.104–1.218)	<0.001	
Cerebral infarction	1.279	3.595 (1.901–6.797)	<0.001	
SBP	0.026	1.026 (1.008–1.045)	0.005	
LDL-C	−0.016	0.984 (0.972–0.995)	0.006	
Current drinker	−0.931	0.394 (0.175–0.886)	0.024	

CI, confidence interval; Cr, creatinine; HDL-C, high-density lipoprotein cholesterol; LDL-C, low-density lipoprotein cholesterol; OR, odds ratio; PVH, periventricular hyperintensities; SBP, systolic blood pressure; 1,5-AG, 1,5-anhydroglucitol.

Model included age, SBP, Cr, HDL-C, LDL-C, 1,5-AG, current drinker, and cerebral infarction.

1 Logistic regression model compared severe PVH versus mild PVH (reference group).

### Predictors of DWMH Severity

Table 3 summarizes participant characteristics by DWMH severity. The logistic regression model included age, SBP, Cr, HDL-C, LDL-C, 1,5-AG, current drinker, and cerebral infarction. We revealed that older age, lower 1,5-AG, elevated SBP, presence of cerebral infarction, and lack of current alcohol use were significant independent predictors of severe DWMH (Table 4).

**Table 3:** Characteristics of participants by DWMH severity

Characteristic	Mild (*n* = 396)	Moderate (*n* = 189)	Severe (*n* = 103)
Male (%)	45.7	42.3	40.8
Age, mean ± SD, years	74.5 ± 6.5	78.8 ± 6.7^1^	80.0 ± 7.3^1^
SBP, mean ± SD, mmHg	134.0 ± 18.1	133.7 ± 17.2	139.9 ± 19.1^1^
DBP, mean ± SD, mmHg	75.0 ± 10.2	74.2 ± 10.8	76.6 ± 10.2
Cr, mean ± SD, mg/dL	0.73 ± 0.22	0.77 ± 0.37	0.82 ± 0.33^1^
HDL-C, mean ± SD, mg/dL	56.4 ± 13.2	57.2 ± 16.1	53.2 ± 12.4^1^
TG, mean ± SD, mg/dL	153.3 ± 103.9	148.7 ± 107.1	144.5 ± 76.4
LDL-C, mean ± SD, mg/dL	101.2 ± 29.4	97.1 ± 27.1	96.3 ± 30.0
1,5-AG, mean ± SD, *μ*g/mL	21.5 ± 9.0	19.0 ± 9.2^1^	18.8 ± 9.2^1^
Cerebral infarction (%)	22.7	37.0^2^	49.5^2^
Hypertension (%)	58.7	70.4^2^	72.8^2^
Impaired glucose tolerance (%)	33.1	34.9	39.8
Hyperlipidemia (%)	32.9	33.9	35.0
Current smoker (%)	9.9	5.7	6.2
Current drinker (%)	35.7	22.3^2^	21.9^2^
mUPDRS, mean ± SD	0.9 ± 2.0	1.1 ± 1.8	1.2 ± 1.6^1^
MMSE, mean ± SD	26.7 ± 3.1	25.1 ± 3.9^1^	24.4 ± 5.0^1^
GDS, mean ± SD	3.0 ± 3.0	3.5 ± 3.4	3.8 ± 3.4
Education, mean ± SD, years	9.8 ± 2.2	9.7 ± 2.3	9.2 ± 2.1

Cr, creatinine; DBP, diastolic blood pressure; DWMH, deep white matter hyperintensities; GDS, Geriatric Depression Scale; HDL-C, high-density lipoprotein cholesterol; LDL-C, low-density lipoprotein cholesterol; mUPDRS, motor component of Unified Parkinson's Disease Rating Scale; MMSE, mini mental state examination; SBP, systolic blood pressure; SD, standard deviation; TG, triglycerides; 1,5-AG, 1,5-anhydroglucitol.

1 *P* < 0.05, Kruskal–Wallis test with post hoc test versus mild.

2 *P* < 0.05, chi-square test versus mild.

**Table 4:** Predictors of severe DWMH^1^

Variable	Regression coefficient	OR (95% CI)	*P*	Predictive accuracy
Intercept	−9.685		<0.001	82.7
Age	0.085	1.088 (1.045–1.133)	<0.001	
Cerebral infarction	1.223	3.396 (1.950–5.915)	<0.001	
1,5-AG	−0.042	0.959 (0.931–0.988)	0.006	
Current drinker	−0.852	0.427 (0.220–0.826)	0.012	
SBP	0.017	1.017 (1.003–1.032)	0.020	

CI, confidence interval; Cr, creatinine; DWMH, deep white matter hyperintensities; HDL-C, high-density lipoprotein cholesterol; LDL-C, low-density lipoprotein cholesterol; OR, odds ratio; SBP, systolic blood pressure; 1,5-AG, 1,5-anhydroglucitol.

Model included age, SBP, Cr, HDL-C, LDL-C, 1,5-AG, current drinker, and cerebral infarction.

1 Logistic regression model compared severe DWMH versus mild DWMH (reference group).

### Predictors of Motor Function

A total of 581 in 688 MRI recorded participants (250 men) were evaluated for motor function by mUPDRS (mean ± SD, 1.0 ± 1.9). There were no significant gender differences. The mUPDRS score was not a significant predictor of severe PVH or DWMH in the multiple regression analyses (data not shown).

### Predictors of Cognitive Function

A total of 660 in 688 MRI recorded participants (290 men) were evaluated using the MMSE (mean ± SD, 25.9 ± 3.8), and women had a significantly higher mean score than men (men, 25.6 ± 3.9; women, 26.2 ± 3.7; *P* = 0.011). Multivariate regression model included PVH, DWMH, age, sex, Cr, HDL-C, LDL-C, cerebral infarction, and years of education. Younger age, female sex, mild DWMH, more years of education, and higher HDL-C levels were significant predictors of better cognitive function (Table 5).

**Table 5:** Predictors of MMSE

Variable	Partial regression coefficient (95%CI)	*P*	Predictor importance	*R*^2^
Intercept	38.105 (34.117 to 42.094)	<0.001		0.235
Age	−0.205 (−0.245 to −0.165)	<0.001	0.754	
DWMH (mild)	0.925 (0.382 to 1.468)	0.001	0.083	
Education	0.219 (0.080 to 0.358)	0.002	0.071	
Sex (men)	−0.739 (−1.257 to −0.222)	0.005	0.058	
HDL-C	0.021 (0.002 to 0.040)	0.034	0.033	

CI, confidence interval; Cr, creatinine; DWMH, deep white matter hyperintensities; HDL-C, high-density lipoprotein cholesterol; LDL-C, low-density lipoprotein cholesterol; MMSE, mini mental state examination; PVH, periventricular hyperintensities.

Model included age, sex, Cr, HDL-C, LDL-C, cerebral infarction, education, PVH, and DWMH.

### Predictors of Depressive Symptoms

A total of 631 in 688 MRI recorded participants (278 men) were evaluated using the GDS. The mean ± SD score for all participants was 3.3 ± 3.2, and there were no significant gender differences. Multivariate regression model included PVH, DWMH, age, sex, LDL-C, 1,5-AG, current smoker and drinker, and years of education. Lower 1,5-AG, lower LDL-C levels, moderate to severe PVH, and no current alcohol use were significant predictors of depressive symptoms (Table 6).

**Table 6:** Predictors of GDS

Variable	Partial regression coefficient (95% CI)	*P*	Predictor importance	*R*^2^
Intercept	2.646 (−0.609 to 5.901)	0.111		0.043
Current drinker	−0.663 (−1.209 to −0.118)	0.017	0.257	
PVH (mild)	−0.601 (−1.152 to −0.049)	0.033	0.206	
LDL-C	−0.009 (−0.018 to −0.001)	0.034	0.204	
1,5-AG	−0.028 (−0.055 to −0.001)	0.044	0.183	
Age	0.035 (−0.003 to 0.073)	0.069	0.149	

CI, confidence interval; DWMH, deep white matter hyperintensities; GDS, Geriatric Depression Scale; LDL-C, low-density lipoprotein cholesterol; PVH, periventricular hyperintensities; 1,5-AG, 1,5-anhydroglucitol.

Model included age, sex, LDL-C, 1,5-AG, current smoker and drinker, education, PVH, and DWMH.

## Discussion

In this population-based study, we investigated the relationship between WMLs, lifestyle factors, and cognitive and motor function and mood in Japanese elderly people. Our study had a high participant rate and included many very elderly in habitants. Independent predictors of severe PVH were older age, cerebral infarction, lower LDL-C levels, elevated BP, and lack of current alcohol use. Independent predictors of severe DWMH were older age, cerebral infarction, lower 1,5-AG levels, and elevated BP, lack of current alcohol use. Individuals with higher global cognitive function were more likely to be younger, female, and have more years of education, higher HDL-C levels, and mild DWMH. Lower levels of depressive symptoms were associated with higher 1,5-AG levels, higher LDL-C levels, current alcohol use, and mild PVH.

In previous studies, age and hypertension were reported to be the most common risk factors for WMLs (Liao et al. 1997; de Leeuw et al. 2002). The risk of cerebrovascular disease was found to be increased in individuals with higher BP and BP fluctuations of greater magnitude (Brickman et al. 2010). Some studies have suggested that white matter hyperintensities are associated with diabetes (Murray et al. 2005; van Harten et al. 2007)whereas others found no association (Bogousslavsky et al. 1987). In this study, hypertension and impaired glucose tolerance were related to DWMH severity. In addition, hypertension was related to PVH severity. Associations of PVH and LDL-C levels were in an unanticipated direction. Hyperlipidemia is a risk factor for vascular disorder (Tirschwell et al. 2004) and statin treatment has demonstrated benefits in stroke prevention and prognosis (Amarenco et al. 2006; Alvarez-Sabin et al. 2007). Consistent with previous findings (Jimenez-Conde et al. 2010; Ichikawa et al. 2012), data in this study indicated that higher LDL-C levels were associated with lower severity of WMLs. The mechanisms of the inverse correlation between LDL-C and PVH are not fully understood, but cholesterol is thought to play important roles in neuron repair and remodeling in the central nervous system (Dietschy and Turley 2001). Mielke et al. indicated that high total cholesterol level in late life is associated with a reduced risk of dementia. Taken together with our result showing higher cholesterol was associated reduction of WMLs, high cholesterol might be associated with better healthy status in late life (Mielke et al. 2005). Moreover, statin treatment may protect the vessels of the brain and increase chances of survival while also being associated with worsening WMLs (Longstreth et al. 2005).

The distinction between PVH and DWMH is of clinical significance as they have been associated with different clinical consequences. DWMH might predominantly disrupt the short association fibers, also known as arcuate U fibers, which connect adjacent cortical areas. PVH probably affects the long association fibers that connect the more distant cortical areas (de Groot et al. 2000). In previous investigations, PVH burden was related to cognitive function (Debette et al. 2007) and a decline in mental processing speed (van den Heuvel et al. 2006). In this study, improved cognitive function was correlated with mild DWMH but was not related to PVH severity. A previous study also found a correlation between DWMH and cognitive function in middle-aged individuals (Soriano-Raya et al. 2012). Differences in study methods and participant races and ages might account for these conflicting findings. Increasing severity of generalized brain atrophy and the presence of cerebral infarcts on MRI are associated with a steeper decline in cognitive function (Prins et al. 2005). Therefore, further investigation is necessary to examine the connections between cognitive function and brain atrophy.

Some authors have found a correlation between depression and WMLs (Iidaka et al. 1996; Jorm et al. 2005; Krishnan et al. 2006; Herrmann et al. 2008). WMLs are caused by cerebrovascular disease and disrupted fiber tracts within frontostriatal circuits. Because of the involvement of frontostriatal circuits in the regulation of mood, disruption of these circuits may lead to a disconnection syndrome (Herrmann et al. 2008). WMLs are believed to be primarily caused by hypoperfusion and arteriosclerosis (Liao et al. 1997). Decline in total cerebral blood flow is associated with an increase in the volume of PVH but not DWMH (ten Dam et al. 2007). PVH may be affected by longer term vascular disorder. Our finding of a relationship between depressive symptoms and PVH supports the vascular hypothesis of depression.

This study has some limitations that should be mentioned. The visual rating scale for assessing WMLs used in this study may differ from quantitative volumetric methods. However, because significant agreement between the Fazekas scale and quantitative volumetric measurement has been shown (Kapeller et al. 2003), the method used to assess WMLs is not expected to affect the findings. The cross-sectional nature of this study precludes conclusions about causality. In addition, this study did not assess the treatments participants received over time, which may have affected results. For example, a previous study found that individuals who used antihypertensive medication and had controlled BP had a reduced risk of severe WMLs (Dufouil et al. 2001; de Leeuw et al. 2002). Both atrophic and ischemic imaging changes are driven by altered glycemic and BP control beginning in midlife (Knopman et al. 2011). We evaluated BP values and clinical biochemistry parameters, but did not include treatment and response to treatment in our models. Although efforts to study regional white matter changes have been initiated, future investigations should seek to define longitudinal relationships between WMLs and other factors.

In conclusion, investigators have begun to take notice of associations between hypertension, glucose intolerance, depression, and cognitive decline. Our population-based study indicated that WMLs in the elderly were related to hypertension and impaired glucose tolerance, and that the severity of WMLs was associated with cognitive function and mood.
